# Strengthening the quality and safety of community mental health care for children and young people: a critical review of innovations from low- and middle-income countries

**DOI:** 10.3389/frhs.2026.1761607

**Published:** 2026-04-29

**Authors:** Eleanor Chatburn, Charmaine Natasha Nyakonda, Lars Dumke, Jennifer Hall, Jean Anne Heng, Joseph Kalisa, Rob Kidney, Elewani Charity Mamathuba, Shobhana Nagraj, Nicholas Pang, Giovanni Salum, Faith Martin

**Affiliations:** 1 Centre for Child, Adolescent and Family Research, Department of Psychology, University of Cambridge, Cambridge, United Kingdom; 2 School of Psychology and Sport Sciences, Anglia Ruskin University, Cambridge, United Kingdom; 3 Department of Clinical Psychology and Psychological Therapies, Norwich Medical School, Norwich, United Kingdom; 4 Grassroot Soccer (GRS), Hanover, NH, United States; 5 WHO Office on Quality of Care and Patient Safety Office, Athens, Greece; 6 Department of Psychiatry and Psychotherapy, University Medical Center Hamburg-Eppendorf, Hamburg, Germany; 7 WHO Office on Quality of Care and Patient Safety Office in Athens, WHO Regional Office for Europe, Athens, Greece; 8 Centre for Mental Health, University of Rwanda, Kigali, Rwanda; 9 Department of Public Health, Aarhus University, Aarhus, Denmark; 10 Stavros Niarchos Foundation (SNF) Global Center for Child and Adolescent Mental Health at the Child Mind Institute, New York, NY, United States; 11 Independent, Gauteng, South Africa; 12 Department of Public Health and Primary Care, University of Cambridge, Cambridge, United Kingdom; 13 East London NHS Foundation Trust, London, United Kingdom; 14 Faculty of Medicine and Health Sciences, Universiti Malaysia Sabah, Kota Kinabalu, Malaysia; 15 Department of Psychology, University of Bath, Bath, United Kingdom

**Keywords:** global mental health, alternative models of access, developing quality standards, peer and near-peer approaches, socio-economic interventions, task-sharing, child and youth mental health

## Abstract

Improving the quality and safety of community mental health care for children and young people (CYP) is a global public health priority. Mental health problems affect 10%–20% of CYP globally. While many high and low- and middle-income countries (LMICs) face significant structural barriers to accessing safe, person-centred mental health services, there have been innovative developments in service delivery emerging from low resource settings that are pertinent to address quality and safety of CYP mental health services globally. In this paper, we critically review emerging evidence from these contexts to highlight best practices and innovations. We focus on six key domains: task-sharing with non-specialists including peer and near-peer approaches; socio-economic interventions; alternative models of access; advances in routine outcome measurement; developing quality standards; and digital interventions. Through three case studies from Malaysia, Rwanda, and South Africa and other contexts within Sub Saharan Africa and high-income countries (HICs), we demonstrate how locally responsive, contextually appropriate solutions can strengthen mental health services in community settings. We argue for reciprocal co-production in global mental health, positioning LMICs as sources of innovation rather than passive recipients of external expertise. We conclude by discussing how these innovations offer transferable lessons for all contexts and identifying pathways to support their sustainability and scalability.

## Introduction

Improving the quality and safety of mental health care is a pressing priority in low- and middle-income countries (LMICs), with this imperative arguably even more pertinent for children and young people (CYP)^1^. The majority of the world's CYP live in LMICs, with mental health disorders impacting 10%–20% of this population (1). Despite this significant burden, CYP face significant barriers to accessing appropriate services (2–4), due in part to stark inequalities in service provision, workforce limitations, and inadequate policy coverage (5). These disparities are compounded by broader structural and systemic challenges, such as poverty, health inequalities, and shortages in professional mental health workforce capacity (6). Further to this, LMICs with postcolonial legacies face the complex challenges of transplantation of Western-centric models with limited cultural adaptation or local collaboration (7, 8). However, the majority of existing work has focused on topics relating to access, while the core domains of quality and safety of mental health services in LMICs remain under-addressed (9). This critical review seeks to reframe the discussion by (1) positioning both quality and patient safety as central priorities and (2) showcasing culturally sensitive innovations and considerations emerging from LMICs that have potential to inform global mental health.

## Quality and safety of mental health services

Quality of mental health care is defined as “a measure of whether services increase the likelihood of desired mental health outcomes and are consistent with current evidence-based practice” (10). There are six domains of quality of care: equity, person-centredness, effectiveness, efficiency, integration, and timeliness of service delivery (11). Many individuals with mental health problems are not able to access the help they need. Challenges to the provision of high-quality mental health care include a lack of funding, scarce workforce, a lack of services, human rights abuses (12) and a lack of standardised approaches to measuring and improving quality of mental health care (13). With clear evidence that the impact of CYP not receiving timely and adequate mental health support extends far into adulthood with considerable socio-economic consequences (14), the WHO has identified the need to improve the quality of mental health care for CYP as a top priority (15, 16).

Patient safety is defined as “the absence of preventable harm to a patient and reduction of risk of unnecessary harm associated with health care to an acceptable minimum” and is recognised as an ethical imperative (17). In mental health care generally, a focus on improving patient safety may encompass the creation of safer health systems and monitoring metrics, use of harm reduction strategies, and better measurement of physical and psychological iatrogenic harms (18). In community-based mental health care^2^, safety challenges include risks related to suicide, intimate partner violence, child abuse, and iatrogenic harms (21). There are a number of environmental drivers that can impact suicide and patient safety in low-resource contexts, including poverty, housing instability, and sleep disturbance (22, 23). While such epidemiological risk factors continue to be an important area of research, the present review examines innovations in service organisation and delivery models.

Both high-income countries (HICs) and LMICs face barriers in improving community mental health care for vulnerable children and young people, with challenges compounded in LMIC settings by systemic and structural barriers outlined earlier. While these challenges are well documented, insufficient attention has been given to the role of innovation and novel models of care, community asset-based approaches, the use of creative therapies, and technological advancements to improving quality and safety of mental health services in low resource settings globally. Such emerging approaches from LMICs offer valuable learning opportunities across all contexts, yet we lack platforms for sharing these practices.

This paper thus provides a non-systematic critical review of such innovations, drawing on our experience as researchers and practitioners. We highlight six domains where LMICs have generated promising practices: task-sharing including peer and near-peer approaches, socio-economic interventions, rethinking access, defining quality standards, improving routine outcome measurement, and digital strategies. These areas were selected because together they represent the key system levers required to expand equitable, high-quality mental health care for CYP in resource-constrained contexts. They encompass complementary dimensions of service innovation: who delivers care (task-sharing approaches), how young people are supported to engage and remain in care (socio-economic interventions, rethinking access), and how the safety, quality, and sustainability of services are ensured (quality standards, outcome measurement, and digital strategies). We focus on these domains because they reflect areas where LMIC actors have developed particularly rich, context-responsive practices that are now informing global thinking on implementation. Where possible, we present case studies demonstrating how locally led solutions across these domains can collectively strengthen the accessibility, acceptability, and effectiveness of youth mental health services.

## Task-sharing approaches to improve community mental healthcare

There is a workforce crisis in child and youth mental health services, with inequity most evident in LMICs where there are only 0.05 CYP mental health workers per 100,000 population (0–19 years old) compared to 4.5 in high-income countries (5). The problem is particularly acute at the community level, with only 46% of low-income countries and LMIC countries reporting community mental health outpatient services for this population compared to 50% of Upper Middle Income Countries and 75% of HIC (12). Task-sharing has emerged as a key approach to addressing treatment gaps and workforce shortages. It involves the systematic redistribution of healthcare tasks from higher- to lower-trained health workers with appropriate training and supervision (4, 24). A range of models have been developed, including those using community health workers, peer supporters, and specialized lay providers across home, community, outpatient clinic, and school settings. Systematic reviews suggest that psychological treatments for common mental health problems delivered by non-specialist providers (including lay counsellors, teachers, and peers) are acceptable, feasible, effective, and affordable (25, 26). However, meta-analytic evidence indicates that youth psychotherapies delivered by lay providers in LMICs, while effective, are outperformed by interventions delivered by professional clinicians (27). Even so, Venturo-Conerly and colleagues argue that the more scalable and cost-effective nature of lay provision means they may still have a valuable role from a public health perspective, though comparative effectiveness trials are urgently needed.

In LMICs, youth access is often further limited by barriers including stigma and restricted mental health information (28–30), alongside low confidence in mental health professionals and limited mental health literacy (31). A recent systematic review of 24 studies found that young people prefer peer and near-peer mental health support workers, who offer relatable lived experience, culturally appropriate spaces, and judgement-free authentic support (32). This is notable in light of findings that show the relationship young people have with intervention providers influences their help-seeking behaviours and engagement with mental health services (33). Peer and near-peer task-sharing interventions, where youth support CYP from the same communities, have emerged as scalable solutions to address this treatment gap (34). These “asset-based” community approaches invert the deficit model by amplifying existing community strengths and resources that promote well-being (35), as exemplified by the work of Grassroot Soccer (see Table 1).

**Table 1:** Community case study 1.

Organisation: Grassroot Soccer
Area of innovation: Near-peer approaches
Context: Young people (10–24 years) in Ethiopia, Kenya, Malawi, Nigeria, South Africa, Zambia, Zimbabwe, Scotland, United States of America, Rwanda, DRC, Ghana, Uganda.
Description: Grassroot Soccer (GRS) began as a youth health initiative in Sub-Saharan Africa using the universal appeal of soccer to connect with young people and improve access to youth-friendly services that promote wellbeing and healthy behaviours. Today, GRS operates across twelve countries, delivering community-based mental health programmes and digital initiatives in partnership with local organisations. Its signature mental health model, *MindSKILLZ*, is led by near-peer mentors (“Coaches,” aged 18–35) who come from the same communities as participants. Coaches use soccer as a hook to foster trust, encourage discussion of mental health, and foster resilience, healthy behaviours, and strong support networks.
The GRS approach is youth-driven and strengths-based: young people and local communities shape programmes through feedback and discussion to ensure accessibility, cultural relevance, and sustainability. Coaches themselves benefit from paid facilitator roles with wellness support and opportunities for professional development, strengthening both personal and community capacity. GRS’ signature SKILLZ programming has consistently demonstrated effectiveness in improving youth health knowledge, building resilience and self-efficacy, and increasing uptake of health services (36–39). A pilot study of *MindSKILLZ* in Kenya further showed promising results: the proportion of participants reporting poor mental wellbeing and depressive symptoms decreased by 46% over the course of the intervention, alongside modest improvements in stigmatizing beliefs, mental health knowledge, attitudes, and care-seeking behaviours (40). The programme was highly acceptable, with over 90% of participants giving the highest possible rating. Participants also reported secondary benefits, including improved school performance and the sharing of mental health information with family and friends. This initiative illustrates how a culturally resonant, near-peer-led model can reframe youth mental health support as accessible, empowering, and embedded in everyday community life. Furthermore, in adapting an approach first developed in Sub-Saharan Africa for high-income countries such as Scotland and the United States, GRS’ work shows that community-led innovations from the Global South can strengthen youth mental health programming globally. This reverse innovation challenges the traditional one-way flow of solutions and demonstrates that foundational elements of mental health promotion such as coping skills and resilience are universal and can transcend geographic and cultural boundaries.

However, task-sharing is not without challenges. Multiple contextual factors must be considered to ensure acceptability and feasibility, with systematic reviews highlighting concerns including burnout, lack of emotional support, threats to personal safety of female workers, difficulties managing boundaries and confidentiality, lack of career progression, and insufficient supervision (41). As youth lay providers can experience their own mental health challenges and caregiver fatigue, comprehensive wellness support is critical to programme success (42). Research gaps remain around optimal training and supervision models to ensure task-sharing enhances rather than compromises quality care, as well as implementation strategies necessary to sustain and embed task-sharing in health systems (43, 44). Addressing these quality, safety, and equity concerns (45) is essential to realising task-sharing's potential as a scalable solution for CYP mental health in resource-constrained settings.

Emerging evidence from youth-led programmes such as Zvandiri and the Friendship Bench highlights how structured supervision and competency-based training can protect both peer supporters and clients. In Zimbabwe, Zvandiri's Community Adolescent Treatment Supporters, young people living with HIV have been trained to deliver evidence-based psychosocial and mental-health interventions, including problem-solving therapy for common mental disorders and adherence-linked emotional support (46, 47). These studies show benefits for young people's mental health and wellbeing, while also identifying risks for peer supporters related to emotional burden, boundary management, and status disclosure (48). Integration of competency frameworks such as EQUIP into peer-training systems (49) and the provision of ongoing mentoring and wellness support represent key innovations to safeguard patient safety and sustain quality within youth task-sharing models.

## Socio-economic interventions

Socio-economic interventions are increasingly recognised as vital components of mental health systems, particularly in LMICs where mental health challenges are linked to socio-economic disadvantage, reduced productivity, and increased healthcare costs (50, 51). Patient safety in this context can be conceptualised as addressing system-level issues, such as lifting young people out of harmful living conditions where they would otherwise continue to be at risk of adverse outcomes. Integrating economic empowerment interventions into mental health policy is especially important for children and young people, where early action delivers long-term benefits (50). These include cash transfers, employment support, and debt relief to reduce financial stress, improve education access, and strengthen family dynamics.

Evidence in this area remains mixed. Cash transfer programmes have been shown to significantly reduce internalising problems in young people including depression and anxiety (52, 53), while imposing conditions (e.g., based on vaccination or school attendance) that exclude the most vulnerable who may be unable to meet requirements (54). Some evidence also suggests that cash transfers can be linked to negative effects on girls’ mental health especially (53). More accessible alternatives include savings-led initiatives such as Village Savings and Loan Associations (VSLAs), common across many sub-Saharan Africa and other LMIC contexts. VSLAs buffer against risk factors for mental health difficulties by increasing economic status, improving quality of life, reducing engagement in hazardous labour, and enhancing community trust (55, 56). While their role in “treating” mental health difficulties remains unclear, the peer-support organisation OPROMAMER provides a notable exception: a complex, multi-level, grassroots intervention in Rwanda with an economic component supporting young people and adults with serious mental health problems (see Table 2).

**Table 2:** Community case study 2.

Organisation: OPROMAMER (Organisation pour la Promotion et la solidarité des Malades et Handicapés Mentaux au Rwanda)
Area of innovation: Socio-economic interventions
Context: Run for and by people with a range of psychiatric diagnoses and experiences, and their families, in Rwanda.
Description: OPROMAMER is a friendship and solidarity peer support organisation that grew from a two-member group in a psychiatric ward in 2012 at Ndera Neuropsychiatric Hospital to a national organisation providing 29 peer support groups across 21 districts of Rwanda. The OPROMAMER model is multi-level, with each small support group operating independently and engaging in activities such as farming, solidarity initiatives (helping members access health services like medical insurance), and offering moral, financial support (through the savings and loan initiatives). The economic activities provide direct support via food and opportunities for trade, and members describe a direct impact on their sense of self-worth. Groups offer social support both in times of difficulty and joy, such as attending wedding celebrations, or burial ceremonies for members who have lost loved ones. Such acts offer alternative stories of strength, dignity, and contribution. OPROMAMER provides a collective voice, addressing the high levels of stigma faced by individuals receiving psychiatric and psychological support in Rwanda (57). This collective voice helps in reshaping how mental health difficulties are understood by individuals, family members and communities, inviting a collective rather than individual response to suffering. Within each local support group (around 31 members), people engage in acts of resistance to social injustices, psychological distress, and economic hardships. Lived experiences and local knowledge are shared to support one another. In situations where members taking medications have difficulty accessing them, OPROMAMER partners with hospitals to invite outreach programmes, and may designate a group member to collect and transport medication for others, reducing both logistical and financial barriers. The OPROMAMER model centres solidarity and shared meaning, rather than isolation, in responding to mental health challenges and shifts the focus from individual pathology to shared social experiences. The organisation's work demonstrates that mental health challenges must not solely be addressed through clinical and Eurocentric frameworks (58).

Multi-component interventions combining economic empowerment with CBT, counselling, or psychoeducation show effectiveness in reducing depression and anxiety symptoms for vulnerable young people (59). However, economic components have typically been conceptualised as means to improve education or healthcare access rather than as active therapeutic components themselves. This requires reconceptualisation of economic interventions as therapeutic components. Economic interventions reduce exposure to socio-economic conditions that trigger and perpetuate poor mental health, particularly for vulnerable children and young people in LMICs experiencing multiple disadvantages, thereby extending the concept of patient safety beyond traditional clinical parameters. Further research is needed to map how economic interventions act through both risk reduction and therapeutic benefits via psychosocial mechanisms such as boosting identity formation and self-efficacy. Embedding these strategies within mental health systems represents an evidence-based and ethical imperative for advancing global mental health equity.

## Rethinking access

Access is a foundational pillar of quality mental health care and remains a pressing challenge in community CYP mental health care in LMICs (2), particularly for families living in rural, under-resourced settings who face transportation, resource, linguistic, legal, and attitudinal barriers (60). The model of traditional clinic-based services arguably presents a problematic trade-off: in return for marginally higher quality, better-resourced healthcare in tertiary centres, patients are forced to travel for hours, resulting in systematic and iatrogenic creation of factors contributing to dropout and non-adherence. Alternative models of service delivery are being explored, including school-based delivery and the task-sharing and peer-based approaches discussed earlier (60). Here, we draw inspiration from the on-demand economy to propose reframing access through principles of responsiveness, convenience, and logistical efficiency. Rather than investing heavily in fixed infrastructure as a means of increasing access, innovative LMIC services mobilise teams and services according to local population needs, thereby improving cost-effectiveness while reducing care delays. This decentralised approach enhances reach while aligning with health economic realities in contexts where establishing new facilities is neither feasible nor sustainable. A case study outlining innovations in CYP psychiatry community care delivery in Malaysia is presented in Table 3. Such service innovations redefine access not merely as a physical location but as adaptability, flexibility, and responsiveness to population needs, ultimately enhancing quality and safety of community mental health services by ensuring timely and sustained engagement with care.

**Table 3:** Community case study 3.

Organisation: Hospital Universiti Malaysia Sabah
Area of innovation: Psychiatric community care delivery models
Context: Borneo rural population in Malaysia
Description: Several innovative psychiatric care delivery models have been pioneered in Sabah, a state on Bornean Malaysia. One such model, CHAMPS (Child and Adolescent Mental and Physical Health Services, uses mobile, “pop-up” multidisciplinary clinics. These integrated, “one-stop-shops” are designed to deliver assessments and treatments in a single visit. A multidisciplinary team of speech therapists, occupational therapists, physiotherapists, clinical psychologists, general doctors, psychiatrists and social workers organise multiday programmes in remote districts. This model reframes access not as a geographic limitation, but as an implementation challenge: when communities cannot come to the health system, the health system must go to them. An extension of this principle is seen in a healthcare delivery model piloted by Hospital Universiti Malaysia Sabah (HUMS) in Sabah, where healthcare is brought directly to patients in homes, schools, or other community hubs. For instance, HUMS pioneered the practice of psychiatrists rotating to primary care clinics in remote areas, providing tertiary care in the Malaysian equivalent of a remote island general practice once a month. Evaluation has shown that this approach greatly reduces gaps in care delivery whilst upskilling general practice clinics to manage less complex psychiatric cases more effectively. Moreover, with the inclusion of clinical year medical students on each visit, this model also teaches the next generation of doctors how to task shift and “Uberise” better, allowing for more equitable distribution and more efficient use of limited medical resources at each level.

## Defining quality standards

Care inequities and inconsistencies for CYP mental health are observed at all levels of the health system globally. Higher staffing ratios have been linked to higher quality of care and patient safety more broadly in health (61). An initial step to reduce care inequities and inconsistencies is to develop a shared definition of what high-quality care looks like (13). Service-level quality standards can define how care should be received and delivered, by whom and how (62). Treatment guidelines and protocols can determine what evidence-based treatment individuals should receive (62). For CYP mental health, these tools provide an opportunity to standardise the quality of care received, independent of location or time. Shared definitions of high-quality care should be co-produced with inputs from those with both lived and professional experience of mental health services (63, 64). However, there is a lack of national quality standards and their evaluation globally for CYP mental health services, with these only being available in a few high-income countries (65). Evidence on how best to improve the quality of CYP mental health care is also lacking, with existing research being focused on high-income countries (66).

In response to the need to standardise care quality (67), the WHO Regional Office for Europe has recently developed quality standards for CYP mental health services (16). Whilst they are applicable across all income levels, their implementation will vary greatly depending on the resources in the country. To facilitate implementation and quality improvement, it is essential to work closely with implementers, such as policy makers and service providers, and to co-develop tools and resources both for implementation and for measuring and evaluating change. Having quality standards which apply across multiple countries enables the identification of gaps and solutions in quality of care for marginalized populations, such as forcibly displaced children and young people (68). Such standards also allow for joint problem solving, sharing of resources (e.g., on which indicators to use to measure the quality standards) and sharing of innovations to improve care quality.

## Improving routine outcome measurement

The use of standardised data in clinical practice including routine outcome measures (ROMs) has great potential for improving the quality and safety of community CYP mental health treatments and services. Specifically, ROMs can provide a structured way to identify early signs of deterioration, monitor clinical progress, adjust care plans in a timely manner, evaluate treatment effectiveness, and reduce the risk of iatrogenic harm (69, 70). A number of specially tailored measures exist for tracking treatment outcomes in children and young people, and a useful online directory is hosted by the Anna Freud, London [see (71)]. As the majority of these tools were developed and normed on samples from industrialised Western contexts, a major priority in global CYP mental health research is the cultural validation and translation of tools for diverse LMIC contexts (72). One example of successful cultural validation of an outcome measure in routine care settings is a project that tested the Health of the Nation Outcome Scales for Children and Adolescents (HoNOSCA) scale at a youth centre in a public hospital in Nairobi, Kenya. In the first known test of the HoNOSCA in an LMIC setting, Wambua and colleagues (73) found that both the clinician-rated and self-reported versions of the measure were easy to use in an outpatient youth clinic and called for more research to test the measure in larger samples and in different care delivery settings.

Despite their potential, ROM uptake is inconsistent in both HICs and LMICs (74). In low-resource contexts where care is often episodic, informal, or constrained by workforce shortages, the systematic application of ROMs is considerably more difficult. Key challenges include high healthcare worker turnover which prevents ROMs from being meaningfully interpreted, use of traditional paper filing systems which impede systematic data collection and prevent spotting of trends, and overburdened staff lacking time for ROM completion alongside treatment as usual protocols (75, 76). The lack of supportive infrastructure (e.g., digital platforms, trained personnel, analytic support) disproportionately affects rural settings and can reinforce urban-rural disparities in outcome-based quality monitoring (77, 78).

Successful implementation depends not only on access to digital tools but also on service delivery models that allow for repeated, structured clinical contact (79). Structural demands can mean that ROM implementation is inconsistent or absent despite good intentions. Therefore, while ROMs hold significant promise for improving care quality and safety, their meaningful implementation in low-resource contexts demands pragmatic adaptations that account for local infrastructure constraints and service delivery realities in order to ensure that children and youth are able to benefit from systematic outcome monitoring.

## Digital innovations

Rapid developments in digital technology, particularly widespread personal device usage, present opportunities to deliver mental health care at an unprecedented scale to vulnerable CYP populations. In low-resourced contexts, digital delivery offers acceptable, non-stigmatising, and time-efficient care, particularly for families with limited availability for in-person services. Recent LMIC innovations include chatbots, interactive voice response (IVR), personalised messaging (e.g., WhatsApp), and online single-session interventions. For example, Grassroot Soccer tested a gamified IVR version of their MindSKILLZ youth intervention (see Table 1) in six African countries (80). Keyan et al. (81) used human-centred design to create a ten-session chatbot-delivered psychological intervention for young people across Pakistan, West Bank/Gaza, Nepal, Jamaica, and South Africa. A recent review of chatbot-assisted parenting programmes concluded that while promising, large-scale evaluations and adaptation for LMICs are still needed (82).

Such rapid growth in the digital mental health market has raised concerns about evidence strength and the quality and safety of accessible interventions. LMIC settings in particular face significant implementation challenges relating to digital infrastructure, alongside gaps in research on cultural appropriateness and cost-effectiveness within heterogeneous LMIC populations (83). Digital tools must be culturally adapted and supported by trained personnel to ensure acceptability, efficacy, and safety. Planning for sustainability is equally vital, as attrition among mental health providers can cause even well-intentioned digital initiatives to lose momentum. Other considerations include built-in safety features and functionality to monitor and report any digital harms (84), as well as community involvement in development to ensure interventions align with local needs and preferences, thereby avoiding harm and improving uptake (85). Aligned with this effort, Gonsalves and colleagues have recently produced a set of ten practical recommendations for incorporating co-design approaches into the development and testing of digital mental health interventions for youth in LMICs (86). For digital interventions to reach their potential in scaling mental health care for vulnerable youth, researchers, implementers, and policy makers must proactively attend to quality and safety domains to maximise benefits and minimise harms.

## Discussion

In this review, we highlight creative innovations to strengthen the quality and safety of community mental health care for children and young people, using examples from LMIC settings, that may be generalisable to other global settings. These examples demonstrate that, even in the face of systemic resource constraints, low-cost, contextually appropriate solutions are being developed for improving community-based mental healthcare for CYP. Notably, we show that the conceptualisation of safety itself spans multiple levels of intervention, from peer support and task-sharing to system-wide reforms aimed at reducing socio-economic inequalities. A genuinely multi-sectoral and holistic approach, embedded within socio-ecological frameworks [e.g., (87)], will be required to advance patient safety in CYP mental health care, addressing both epidemiological risk factors such as poverty, housing instability, and sleep disturbances (22, 23), and the service delivery innovations that strengthen system capacity to support vulnerable populations.

Aligned with this wider movement, reciprocal co-production offers a promising model for how innovations diffuse across contexts. By reversing established hierarchies in traditional care systems, this approach positions LMICs as sources of innovation rather than passive recipients of external expertise (88). As shown in our case example from Grassroot Soccer, first developed in Sub-Saharan Africa and subsequently adapted for Scotland and the United States, some of the most creative and culturally resonant community-led solutions to youth mental health challenges are emerging from LMICs themselves. Indeed, the Coalition for Scaling Mental Health's White Paper (89) makes a compelling case for such a reversed learning paradigm, underscoring how LMIC innovations can inform practice in HICs, particularly with respect to accessibility, cultural responsiveness, and community engagement.

A key challenge, however, lies in ensuring that these innovations are not only highlighted but also sustained and adapted. The question of scalability provides an important avenue for future research: how can grassroots, community-driven interventions be expanded without losing the authenticity and local ownership that underpin their effectiveness? Many promising models risk dilution or distortion when incorporated into formal health systems. As such, greater investment in implementation-focused research is urgently needed to identify strategies for scaling that preserve local ownership while enabling wider uptake. Adaptation to other settings should similarly be guided by principles of contextual sensitivity and co-production, rather than mere transplantation.

Taken together, our review highlights the urgent need for more investment, research, and coordinated action to define and strengthen patient safety within CYP mental health. By reframing quality and safety as central priorities and showcasing innovations from LMICs, we aim to foreground pathways towards safer, more equitable, and culturally grounded care for children and young people worldwide.

## Footnotes

^1^By “children”, we refer to people aged 0–18 years and by “young people”, we refer to people aged 15–24 years.

^2^While inpatient CYP psychiatry is not the focus of this present review, we acknowledge that there are considerable additional challenges associated with psychiatric hospital reform. Patient safety in inpatient psychiatry is a neglected issue globally (19). Many institutions are affected by human rights violations, poor conditions, understaffing, and unsafe practices, and these safety issues are more acute in low-resource contexts (20).
